# Health-related quality of life after pulmonary tuberculosis in South Korea: analysis from the Korea National Health and Nutrition Examination Survey between 2010 and 2018

**DOI:** 10.1186/s12955-021-01833-6

**Published:** 2021-08-09

**Authors:** Sang Hyuk Kim, Hyun Lee, Youlim Kim

**Affiliations:** 1 Division of Pulmonology and Critical Care Medicine, Samsung Medical Center, Department of Medicine, Sungkyunkwan University School of Medicine, Seoul, Korea; 2 Division of Pulmonary Medicine and Allergy, Department of Internal Medicine, College of Medicine, Hanyang University, Seoul, Korea; 3 Division of Pulmonary, Allergy and Critical Care Medicine, Department of Internal Medicine, Hallym University Chuncheon Sacred Heart Hospital, 77, Sakju-ro, Chuncheon-si, Gangwon-do 200-704 Republic of Korea

**Keywords:** Tuberculosis, Pulmonary tuberculosis, EQ-5D, Health-related quality of life, Complex survey analysis

## Abstract

**Background:**

Although several studies have reported an association between tuberculosis and health-related quality of life, the change in health-related quality of life after pulmonary tuberculosis has been rarely studied. The purpose of this study was to investigate the effect of past history of pulmonary tuberculosis on health-related quality of life using a nationwide, cross-sectional, observational study in Korea.

**Methods:**

Among 72,751 people selected using a stratified multi-stage sampling method, 7260 Korean participants were included using propensity score matching. Past history of pulmonary tuberculosis was defined as a previous diagnosis of pulmonary tuberculosis excluding patients with active pulmonary tuberculosis. The primary outcome, health-related quality of life, was assessed by EQ-5D disutility.

**Results:**

Before matching, the mean EQ-5D of individuals with pulmonary tuberculosis history was lower (0.066 vs. 0.056, *p*: 0.009). However, the difference was nullified after matching (0.066 vs. 0.062, *p* = 0.354). In multivariable Poisson regression analysis, EQ-5D disutility score was not associated with past pulmonary tuberculosis history. In subgroup analysis, past pulmonary tuberculosis history increased odds of low health-related quality of life in young (odds ratio [OR] 1.57, 95% confidence interval [CI] 1.17–2.11, *p* = 0.003), unmarried (OR 1.98, 95% CI 1.05–3.73, *p* = 0.036), or separated patients (OR 1.30, 95% CI 1.02–1.66, *p* = 0.032). Age and marital status were modulating factors on the effect of past pulmonary tuberculosis history on health-related quality of life.

**Conclusions:**

There was no difference in health-related quality of life between individuals with and without past pulmonary tuberculosis history. Young and unmarried groups had increased odds for low health-related quality of life after pulmonary tuberculosis due to modulating effects of age and marital status.

**Supplementary Information:**

The online version contains supplementary material available at 10.1186/s12955-021-01833-6.

## Background

Pulmonary tuberculosis (TB) is an airborne transmitted respiratory disease caused by *Mycobacterium tuberculosis* [1]. Although the incidence of pulmonary TB has decreased with vaccinations and the development of strategies for prevention and treatment, approximately 10 million people are infected with TB, and 1.5 million people died in 2019 worldwide [2]. The prevalence rate of pulmonary TB in South Korea remains high and is the highest among Organization for Economic Co-operation and Development (OECD) countries [3]. In 2019, the Korean government spent 69 billion won on TB prevention and treatment, but 1610 people still died from pulmonary or extrapulmonary TB [4].

According to the World Health Organization (WHO) definition, quality of life is an individual’s perceptions of their position in life in the context of the culture and value systems in which they live and concerning their goals, expectations, standards, and concerns [5]. Health-related quality of life (HRQoL) has been recently suggested as an additional treatment goal for many diseases, including pulmonary TB, a highly contagious disease that requires isolation and long-term treatment [6–8]. These factors can affect HRQoL as well as mental health issues such as depression and anxiety disorder [9, 10]. In addition, WHO has emphasized patient-centered care in the End TB Strategy [11]. Evaluation of HRQoL is essential to achieve the goals of the End TB strategy [12]. Therefore, in the treatment of pulmonary TB, the HRQoL of the patient must be considered in addition to biological management.

Several studies have reported an association between TB infection and decreased HRQoL. Guo et al. showed that active TB significantly impacted patients’ quality of life, and Atif et al. found improved HRQoL at the end of pulmonary TB treatment [13, 14]. Although previous studies have examined the quality of life during pulmonary TB treatment, few have reported the change in HRQoL after pulmonary TB. Therefore, our study aimed to assess HRQoL among individuals with past history of pulmonary TB using data from the Korea National Health and Nutritional Examination Survey (KNHANES), a nationwide, cross-sectional, observational study.

## Methods

### Study population

**Fig. 1:**
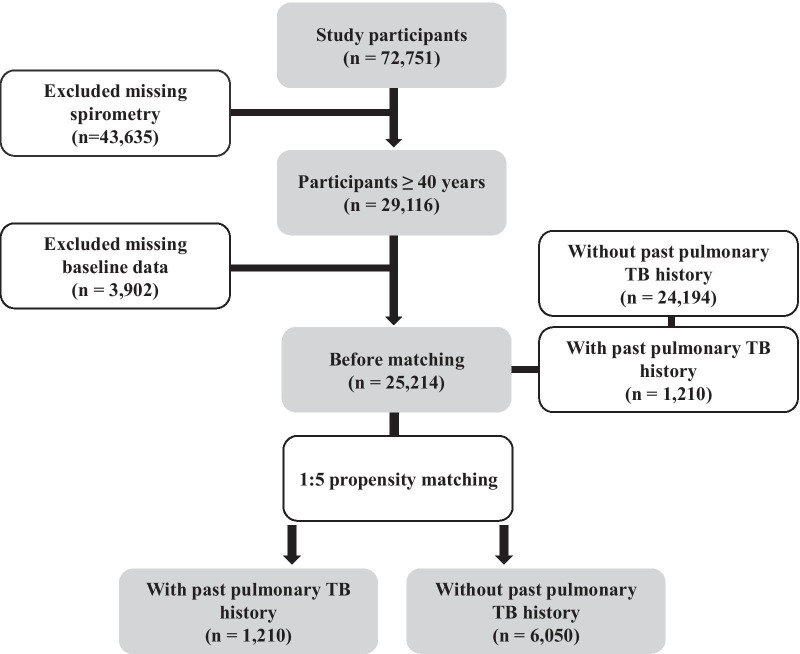
Flow chart of the study. In the KNHANES, spirometry was measured only in participants over 40 years of age

This study used data from KNHANES V (2010–2012), VI (2013–2015), and VII (2016–2018). The KNHANES, conducted by the Korea Centers for Disease Control and Prevention (KCDC), is a nationwide, cross-sectional survey to assess the health and nutritional status of the general population [15]. The study population was selected using a stratified multi-stage sampling method. Of 92,592 participants, 72,751 agreed to participate (participation rate: 78.6%). In the KNHANES, spirometry was assessed only in participants over 40 years of age. Therefore, excluding 43,635 participants without spirometry, 29,116 with spirometry were included as a first step. Of these, 3902 with missing baseline data were excluded. Among the 25,214 remaining participants, 1210 who had experienced pulmonary TB and 6050 without a past pulmonary TB history were selected using 1:5 propensity score matching for age, sex, body mass index (BMI), marital status, smoking status, education, regular exercise, functional expiratory volume in 1 s (FEV_1_), functional vital capacity (FVC), and FEV_1_/FVC. Finally, 7260 participants were included in the final analysis (Fig. 1).

### Past pulmonary tuberculosis history

A standardized interview was conducted by trained staff in the homes of the participants to determine past pulmonary TB history. Past pulmonary TB history was defined as a previous pulmonary TB diagnosis, excluding patients with active pulmonary TB. A previous diagnosis of pulmonary TB was assessed using the question “Have you ever been diagnosed with pulmonary TB by a doctor?” Among the patient group, 1219 participants reported a previous diagnosis of pulmonary TB. Of the 1219 patients, 9 who underwent treatment of active pulmonary TB were excluded. Finally, 1210 participants were defined as those with past pulmonary TB history.

### Health-related quality of life

The HRQoL was assessed using EQ-5D, which consists of the five dimensions of mobility, self-care, usual activities, pain/discomfort, and anxiety/depression. Patient responses to each dimension were scored as follows: one for no problem, two for a moderate problem, and three for a severe problem. The EQ-5D utility scores were calculated by converting response values using the Korean formula for quality of life weighting [16]. The EQ-5D utility score was converted into EQ-5D disutility score (1-EQ-5D utility score) due to the non-normal distribution of EQ-5D utility score.

### Measurements

Income was defined as low for the lowest quartile of monthly household income, intermediate for the second to third quartiles of monthly household income, and high for the highest quartile of monthly household income. Past smoking was defined as those who have smoked more than five cigarettes in their lifetime but currently do not smoke. BMI was calculated by dividing weight by the square of height (kg/m^2^). Lung function test was performed by trained technicians with a spirometer (Vyntus Spiro; Care Fusion, San Diego, CA, USA; or dry rolling seal spirometer Model 2130; Sensormedics Corporation, Yorba Linda, CA, USA), and the best scores of three pre-bronchodilator measurements were recorded. Percent predicted FVC and FEV_1_ were calculated using the predicted values for Korean patients [17]. Blood pressure (BP) was measured using a standard mercury sphygmomanometer (Baumanometer Wall Unit 33 [0850]; Baum Co., Inc., Copiague, NY, USA). Measurements were performed three times, and the average was used. Hypertension (HTN) was defined as systolic BP ≥ 140 mmHg, diastolic BP ≥ 90 mmHg, or treatment with antihypertensive drugs. Blood glucose level was obtained after at least eight hours of fasting and analyzed in a central laboratory (Neodin Medical Institute, Seoul, Korea) using a Hitachi Automatic Analyzer 7600 (Hitachi, Tokyo, Japan). Diabetes mellitus (DM) was defined as fasting glucose ≥ 126 mg/dL and treatment with insulin or oral antidiabetic drugs. Cancer was defined as a previous diagnosis of stomach cancer, liver cancer, lung cancer, or colon cancer. Depressive disorder was defined as a previous diagnosis of major depressive disorder. Regular walking was defined as walking at least five days a week for 30 min or longer. Regular muscle-strengthening exercise was defined as muscle-strengthening exercising at least two days a week. Severe stress was determined based on answer to a self-reported questionnaire item.

### Statistical analysis

Sample weights were used for post-survey adjustment with the ‘survey’ package of R, and participants in the single primary survey unit (PSU) stratum were centered at the sampled grand mean. Before and after propensity score matching, clinical characteristics were compared based on past pulmonary TB history. In the analysis, all variables were categorized, and weight percent (Wt %) was calculated for each. Differences between individuals with and without pulmonary TB history were confirmed using Pearson’s Chi-square test. The EQ-5D disutility according to past pulmonary TB history was assessed using density plots and a design-based t-test. Multivariable Poisson regression analysis was used to analyze the association between past TB history and EQ-5D disutility score because the distribution of EQ-5D disutility score followed the Poisson distribution. Thus, the odds ratio (OR) of multivariable Poisson regression analysis indicated a likelihood of low HRQoL. For analysis, all dimensions were categorized into positive for two or three respondents and negative for one respondent. Multivariable logistic regression analysis was performed to investigate the effect of past pulmonary TB history on each dimension. In subgroup analysis, we analyzed all variables used in multivariable Poisson regression analysis and selected those that showed statistically significant results or that were clinically important. Interaction analysis was performed by including interaction terms in the multivariable Poisson regression analysis. Marital status was analyzed as a factor variable, and other variables were analyzed as ordinal. The *p*-values less than 0.05 were considered statistically significant. All analyses were performed using R version 4.0.3 (R core 172 Team 2020; R Foundation for Statistical Computing, Vienna, Austria).

## Results

**Fig. 2:**
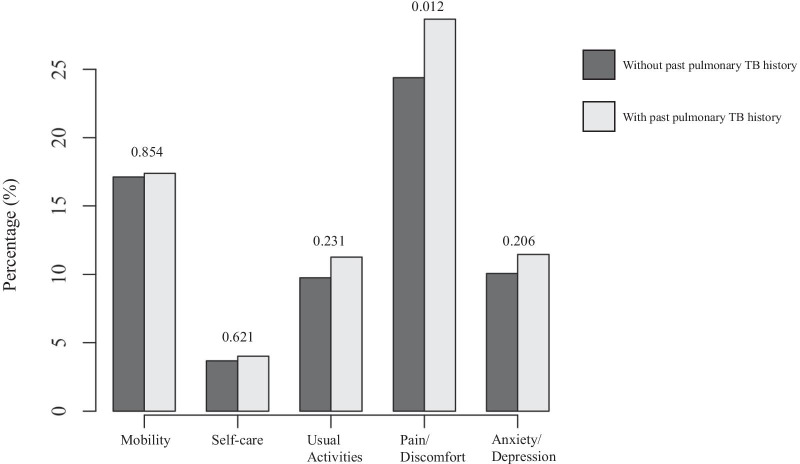
Comparison of weight percent of EQ-5D by past pulmonary TB history. The numbers above bar graphs indicate *p*-value of Pearson’s Chi-square test

For the 25,214 participants, 7260 matched participants were selected using propensity score matching with age, sex, BMI, marital status, smoking status, education, regular exercise, FEV_1_, FVC, and FEV_1_/FVC. Propensity score distributions before and after matching are shown in Additional file 1: Fig. S1. After adjustment for sampling weight, 4,375,915 participants had no past pulmonary TB history, and 749,112 individuals had such a history. Table 1 lists the clinical characteristics of participants according to past pulmonary TB history before and after propensity score matching. Before matching, individuals with past pulmonary TB history were younger with lower BMI compared with those without pulmonary TB history. The proportions of men, divorcees, past smokers, regular muscle exercise, decreased lung function, and HTN were higher in individuals with past pulmonary TB history. However, there were no differences between individuals with and without pulmonary TB history after propensity score matching. As shown in Additional file 2: Fig. S2, the mean EQ-5D disutility scores of individuals with and without past pulmonary TB history were significantly different before matching (0.066 vs. 0.056, *p* = 0.009) but not different after matching (0.066 vs. 0.062, *p* = 0.354). In the dimensional analysis, the proportion of those with pain/discomfort was significantly higher in individuals with past pulmonary TB history, but there were no other differences (Fig. 2).

**Table 1:** Clinical characteristics of the study population before and after matching

	Pre-matched (n = 25,214)			Post-matched (n = 7260)		
	Without past pulmonary TB history (n = 24,004)	With past pulmonary TB history (n = 1210)	*p*-value	Without past pulmonary TB history (n = 6050)	With past pulmonary TB history (n = 1210)	*p*-value
Age (years)			< 0.001*			0.305
40–49	34.22	22.89		24.46	22.89	
50–59	30.90	31.58		27.05	31.58	
60–69	19.95	23.50		24.74	23.50	
≥ 70	14.93	22.03		23.75	22.03	
Sex			< 0.001*			0.757
Male	47.16	58.64		59.24	58.64	
Female	52.84	41.36		40.76	41.36	
BMI (kg/m^2^)			< 0.001*			0.135
< 18.5	1.51	3.50		2.65	3.50	
18.5–24.9	60.69	68.89		69.03	68.89	
25–29.9	33.57	24.68		26.16	24.68	
≥ 30	4.23	2.94		2.15	2.94	
Marital status			0.045*			0.564
Married	84.06	83.18		82.22	83.18	
Widowed/Separated	8.50	8.08		9.19	8.08	
Divorced	4.49	6.4		5.17	6.4	
Unmarried	2.95	2.34		3.42	2.34	
Income			0.803			0.822
Low	23.97	23.87		24.28	23.87	
Intermediate	50.72	51.73		51.20	51.73	
High	25.30	24.41		24.52	24.41	
Education			0.055			0.383
Elementary school graduate	24.55	28.13		26.44	28.13	
Middle/High school graduate	48.22	46.14		47.03	46.14	
College graduate	27.23	25.74		26.53	25.74	
Smoking status			< 0.001*			0.949
Non-smoker	56.82	49.07		49.77	49.07	
Past smoker	22.74	30.46		29.82	30.46	
Current smoker	20.44	20.47		20.41	20.47	
Physical activity						
Regular walking	37.75	38.42	0.692	39.11	38.42	0.550
Regular muscle exercise	21.19	23.80	0.049	23.85	23.80	0.488
Mental health						
Severe stress	22.83	22.30	0.704	20.09	22.30	0.254
Depressive disorder	4.42	4.62	0.771	3.61	4.62	0.373
Lung function						
FEV_1_ (%-predicted)			< 0.001*			0.949
≥ 80	83.98	65.48		65.71	65.48	
< 80	16.02	34.52		34.29	34.52	
FVC (%-predicted)			< 0.001*			0.545
≥ 80	86.61	75.24		75.86	75.24	
< 80	13.39	24.76		24.14	24.76	
FEV_1_/FVC			< 0.001*			0.876
≥ 0.7	87.48	71.10		70.84	71.10	
< 0.7	12.52	28.90		29.16	28.90	
Comorbidities						
HTN	36.94	40.64	0.030*	41.23	40.64	0.588
DM	13.47	13.15	0.787	15.75	13.15	0.052
Cancer	0.67	0.90	0.368	0.92	0.90	0.814

Participants were matched using propensity score matching with age, sex, BMI, marital status, smoking status, education, regular muscle exercise, FEV1, FVC, and FEV1/FVC. Values are expressed as weight percentage. The* *p*-value < 0.05 indicates significant difference between two groupswas calculated using the design-based t-test

TB, tuberculosis; BMI, body mass index; FEV_1_, forced expiratory volume in 1 s; FVC, forced vital capacity; HTN, hypertension; DM, diabetes mellitus

**Fig. 3:**
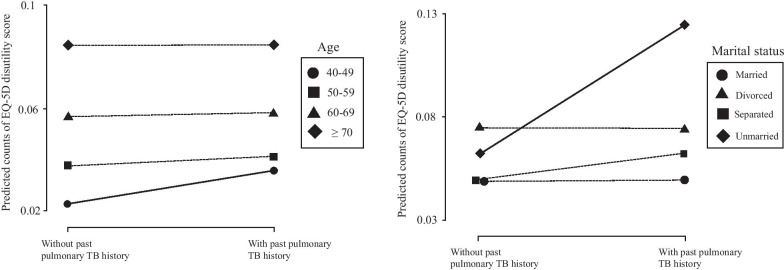
Predicted counts of EQ-5D disutility score according to age and marital status. Solid line indicates positive modulating effect, and dashed line indicates negative modulating effect

We further explored the odds of low HRQoL using multivariable Poisson and logistic regression analysis (Table 2). Among the 5 dimensions, past pulmonary TB history increased odds of pain/discomfort (odds ratio [OR] 1.26, 95% confidence interval [CI] 1.07–1.49, *p* = 0.007). However, the effect of past pulmonary TB history on the EQ-5D disutility score was not significant (OR 1.08, CI 0.97–1.20, *p* = 0.187). In subgroup analysis, a significant odds of low HRQoL was observed in young (OR 1.57, CI 1.17–2.11, *p* = 0.003), separated (OR 1.30, CI 1.20–1.66, *p* = 0.032), or unmarried participants (OR 1.98, CI 1.05–3.73, *p* = 0.036) (Table 3). Interaction between age and marital status also modulated the effect of past pulmonary TB on low HRQoL (*p* for interaction, age = 0.033, unmarried = 0.006). Modulating effects were visualized using an interactive plot shown in Fig. 3.

**Table 2:** The effect of past pulmonary TB history on HRQoL

	EQ-5D disutility score	Mobility	Self-care	Usual activities	Pain/Discomfort	Anxiety/Depression
	OR (95% CI, *P*)	OR (95% CI, *P*)	OR (95% CI, *P*)	OR (95% CI, *P*)	OR (95% CI, *P*)	OR (95% CI, *P*)
Univariate	1.06 (0.94–1.21, 0.345)	1.02 (0.84–1.23, 0.853)	1.10 (0.77–1.56, 0.612)	1.18 (0.91–1.51, 0.210)	1.24 (1.05–1.47, 0.010)	1.16 (0.93–1.44, 0.188)
Model 1	1.08 (0.96–1.22, 0.180)	1.06 (0.87–1.29, 0.566)	1.13 (0.79–1.61, 0.514)	1.23 (0.95–1.59, 0.121)	1.27 (1.07–1.50, 0.005)	1.17 (0.94–1.46, 0.160)
Model 2	1.08 (0.97–1.20, 0.187)	1.06 (0.86–1.30, 0.607)	1.16 (0.80–1.68, 0.162)	1.24 (0.95–1.62, 0.110)	1.26 (1.07–1.49, 0.007)	1.15 (0.91–1.46, 0.230)

ORs and 95% CI were analyzed using multivariable Poisson regression analysis for EQ-5D disutility score and multivariable logistic regression analysis for each dimension

Model 1: adjustment for age, sex, and BMI

Model 2: Further adjustment for marital status, income, education, smoking status, regular walking, regular muscle exercise, severe stress, depressive disorder, FVC, HTN, DM, and cancer

TB, tuberculosis; HRQoL, health-related quality of life; OR, odds ratio; CI, confidence interval; BMI, body mass index; FVC, forced vital capacity; HTN, hypertension; DM, diabetes mellitus

**Table 3:** Subgroup analysis for the effect of past pulmonary TB history on EQ-5D disutility score

Subgroup	EQ-5D disutility score	
	OR (95% CI, *P*)	*P* for interaction
Age		0.033
40–49 (n = 1394)	1.57 (1.17–2.11, 0.003)	
50–59 (n = 1950)	1.08 (0.87–1.34, 0.499)	
60–69 (n = 2184)	1.02 (0.82–1.28, 0.858)	
≥ 70 (n = 1732)	1.00 (0.84–1.19, 0.989)	
Sex		0.815
Male (n = 4066)	1.10 (0.94–1.28, 0.248)	
Female (n = 3194)	1.06 (0.90–1.24, 0.489)	
Marital status		
Married (n = 5948)	1.02 (0.89–1.16, 0.805)	– (Reference)
Separated (n = 736)	1.30 (1.02–1.66, 0.032)	0.067
Divorced (n = 391)	1.08 (0.72–1.61, 0.712)	0.908
Unmarried (n = 185)	1.98 (1.05–3.73, 0.036)	0.006
Income		0.856
Low (n = 1635)	1.06 (0.87–1.29, 0.572)	
Intermediate (n = 3683)	1.02 (0.86–1.21, 0.797)	
High (n = 1942)	1.08 (0.86–1.35, 0.492)	
Education		0.936
Elementary (n = 2012)	1.06 (0.89–1.26, 0.506)	
Middle/high school (n = 3423)	1.14 (0.97–1.34, 0.110)	
College (n = 1825)	0.95 (0.69–1.32, 0.771)	
Smoking status		0.541
Non-smoker (n = 3803)	1.03 (0.90–1.18, 0.665)	
Past smoker (n = 2157)	1.14 (0.90–1.44, 0.276)	
Current smoker (n = 1300)	1.11 (0.85–1.45, 0.426)	
Severe stress		0.972
No (n = 5834)	1.08 (0.95–1.23, 0.248)	
Yes (n = 1426	1.05 (0.84–1.30, 0.681)	
FEV_1_/FVC		0.660
≥ 0.7 (n = 5068)	1.10 (0.96–1.26, 0.180)	
< 0.7 (n = 2192)	1.03 (0.86–1.25, 0.729)	

ORs and 95% CI were analyzed using multivariable Poisson regression analysis adjusted for age, sex, BMI, marital status, income, education, smoking status, regular walking, regular muscle exercise, severe stress, depressive disorder, FVC, HTN, DM, and cancer. The variable used to divide subgroups was excluded from the analysis

TB, tuberculosis; OR, odds ratio; CI, confidence interval; BMI, body mass index; FEV_1_, forced expiratory volume in 1 s; FVC, forced vital capacity; HTN, hypertension; DM, diabetes mellitus

## Discussion

In the present study, even though HRQoL after pulmonary TB in the total study population was not changed, young and unmarried individuals with past pulmonary TB history had an increased odds of low HRQoL due to modulating effects of age and marital status.

Although pulmonary TB is a curable disease, the mental experience associated with treatment can affect patients long-term. Few studies have examined HRQoL after treatment of pulmonary TB. Datta et al. reported that HRQoL was lower in the TB group before treatment but recovered to a level similar to that of the non-TB group after successful treatment [18]. Kastien-Hilka et al. found low HRQoL at the beginning of pulmonary TB treatment, but HRQoL improved significantly during the treatment course [19]. In contrast, Daniels et al. showed low HRQoL, decreased exercise capacity, and reduced lung function after pulmonary TB [20]. However, this might have been due to pulmonary TB's physical sequelae, such as lung function impairment, rather than the experience of pulmonary TB itself. Our study also showed a low HRQoL and decreased lung function in individuals with past pulmonary TB history before propensity score matching, but differences disappeared after matching. We propose that HRQoL can be maintained if patients recover from pulmonary TB without sequelae.

In this study, age and marital status modulated the impact of pulmonary TB history on HRQoL. In particular, the odds of low HRQoL was increased in the young age group (40–49 years old). This result implies that the quality of life of individuals who had an active social life, such as through working and community activities, may be more affected by pulmonary TB. Pulmonary TB, a highly contagious disease, requires isolation precautions and isolates patients from society, negatively affecting mental health [21, 22]. Besides, social stigma around infectious disease was associated with low HRQoL [23, 24]. Thus, young individuals' social life is restricted by pulmonary TB, and limitations of social life remain even after treatment is complete. Decreased lung function after pulmonary TB infection can be another reason for low HRQoL in young individuals. Several studies have reported that pulmonary TB is associated with decreased lung function [25–27]. The discomfort related to lung function impairment might be more evident in young individuals who work regularly. Although spirometric indices were adjusted to similar levels using propensity score matching, decreased lung function can have various causes. Respiratory symptoms can vary depending on the type of chronic lung disease [28, 29]. We speculated that these factors could be associated with low HRQoL after pulmonary TB. Unmarried individuals also were at high odds of low HRQoL associated with past pulmonary TB history. Unmarried persons might be living in single-person households. Family support is an important part of TB treatment and can help lead to favorable treatment outcomes and healthy physical and psychological conditions [30, 31]. This suggests that social support for the unmarried should be attained outside of the family. For this reason, unmarried individuals with pulmonary TB history can experience a significant decrease in HRQoL. The unmarried rate in Korea has been steadily increasing [32]. Therefore, there is a need for adequate community engagement and a social insurance system for individuals in single-person households diagnosed with infectious diseases. Also, caregivers should be aware that there are high-risk groups for low HRQoL even after a successful biological cure, and proper interventions and appropriate advice will be needed.

Our study has several strengths. First, our findings can be generalized and more representative than those of previous reports because we used nationwide multi-year survey data. Most previous studies were small or pilot studies. Second, selection bias was controlled using propensity score matching. As pulmonary TB infection causes various damage, low HRQoL after pulmonary TB infection should be carefully evaluated. We tried to adjust differences in mental and physical conditions following pulmonary TB infection to avoid selection bias. Finally, important sociodemographic factors were included in our analyses. Social factors are important aspects of maintaining the quality of life for individuals with contagious diseases such as TB, human immunodeficiency virus, and coronavirus [30, 33, 34].

There are some limitations to this study. First, past pulmonary TB history was defined based only on participant response. Objective findings suggesting pulmonary TB infection, such as chest x-ray and sputum study results, were not obtained. Thus, the number of respondents with past pulmonary TB history might be underestimated. Second, EQ-5D is not a pulmonary TB–specific HRQoL index, and pulmonary TB is characterized by easy transmission through the air and is more likely in social individuals [6]. Therefore, assessment for social connection is necessary but not included in EQ-5D. Further studies will be needed to develop an HRQOL index specialized for infectious disease, including pulmonary TB, and create a mapping algorithm between a pulmonary tuberculosis non-utility questionnaire that includes social connection and EQ-5D. Third, our study's results should be carefully interpreted as the study was conducted only in Korea, which has a high population density and a national medical insurance system. These circumstances must be considered when practicing medicine in other countries.

## Conclusions

In conclusion, there was no difference in HRQoL between individuals with and without past history of pulmonary TB. However, young age and unmarried groups increased odds for low HRQoL after pulmonary TB infection due to modulating effects of age and marital status.

## Supplementary Information

**Additional file 1: Fig. S1.** Propensity score distribution by past pulmonary tuberculosis history.

**Additional file 2: Fig. S2.** Distribution and weighted mean of EQ-5D disutility score by past pulmonary tuberculosis history. Solid vertical line indicates weighted mean of individuals without past pulmonary tuberculosis, and dashed vertical line indicates weighted mean of individuals with past pulmonary tuberculosis. Weighted means between two groups were compared using a design-based t-test and are presented as p-value.

## Data Availability

Our datasets are available from the corresponding author on reasonable request.

## Abbreviations

TB: Tuberculosis
OECD: Organization for Economic Co-operation and Development
WHO: World Health Organization
HRQoL: Health-related quality of life
KNHANES: Korea National Health and Nutritional Examination Survey
KCDC: Korea Centers for Disease Control and Prevention
BMI: Body mass index
FEV_1_: Forced expiratory volume in 1 s
FVC: Forced vital capacity
BP: Blood pressure
HTN: Hypertension
DM: Diabetes mellitus
PSU: Primary survey unit
OR: Odds ratio
CI: Confidence interval
IRB: Institutional Review Board
