# Supplementary figures and images for: Health-related quality of life after pulmonary tuberculosis in South Korea: analysis from the Korea National Health and Nutrition Examination Survey between 2010 and 2018
Source: Health Qual Life Outcomes. 2021 Aug 9;19:195. doi: 10.1186/s12955-021-01833-6 (PMC8350551; doi:10.1186/s12955-021-01833-6)

Figure S1.

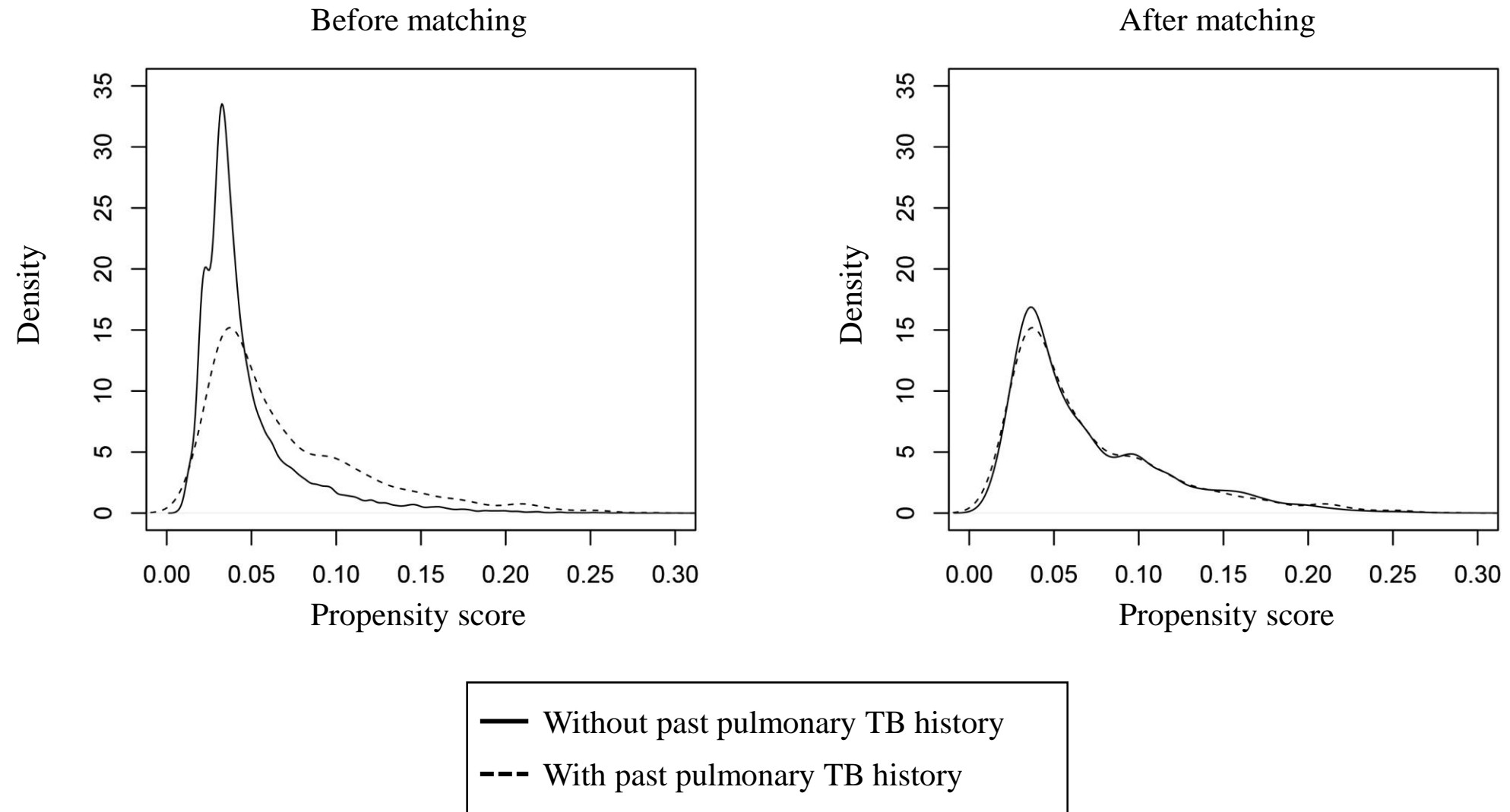

Supplement: Supplementary file 1 — Additional file 1: Fig. S1. Propensity score distribution by past pulmonary tuberculosis history. [file 12955_2021_1833_MOESM1_ESM.pdf]

Figure S2.

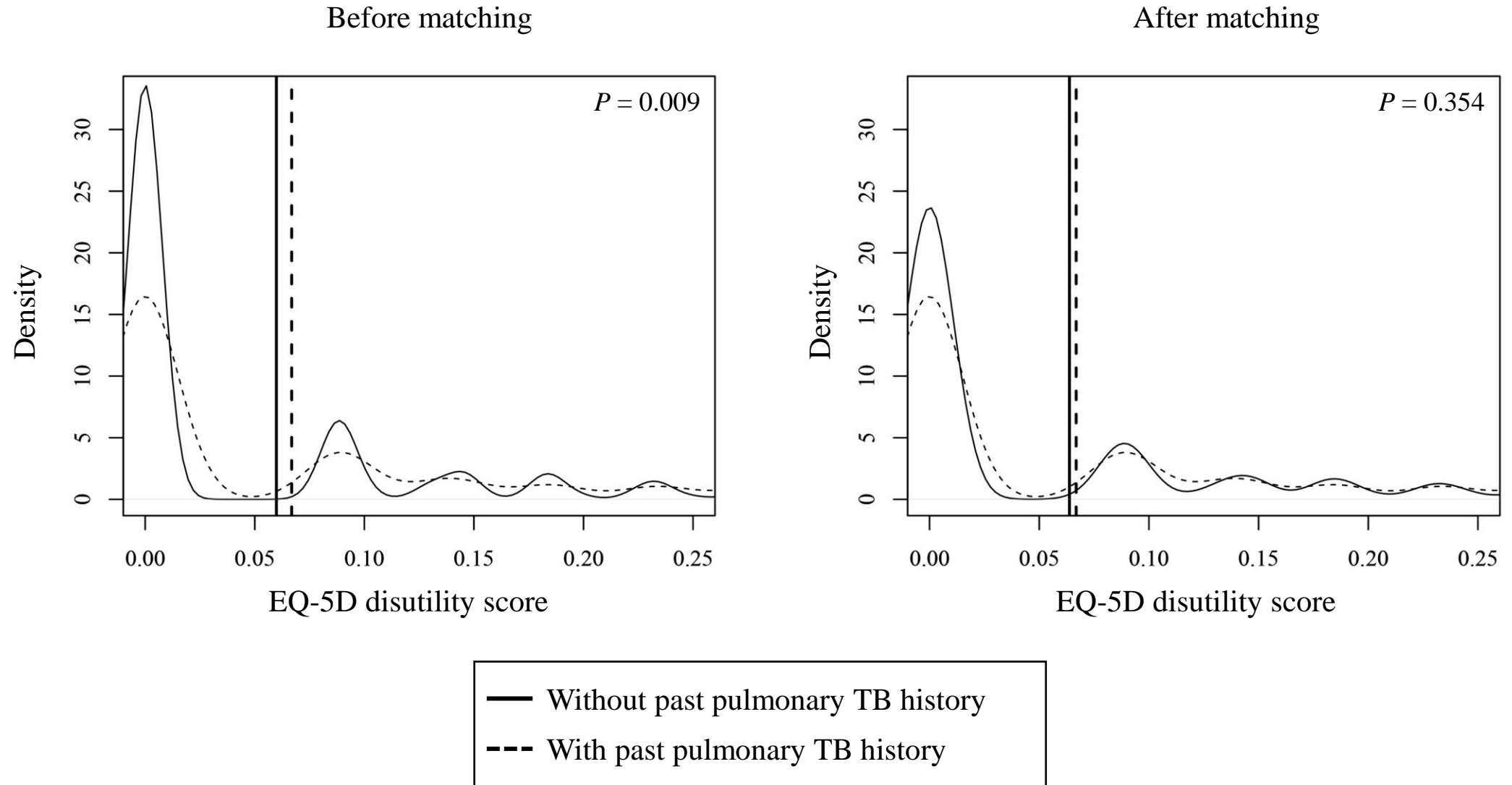

Supplement: Supplementary file 2 — Additional file 2: Fig. S2. Distribution and weighted mean of EQ-5D disutility score by past pulmonary tuberculosis history. Solid vertical line indicates weighted mean of individuals without past pulmonary tuberculosis, and dashed vertical line indicates weighted mean of individuals with past pulmonary tuberculosis. Weighted means between two groups were compared using a design-based t-test and are presented as p-value. [file 12955_2021_1833_MOESM2_ESM.pdf]
